# Disseminated BCG infection in a child with multifocal osteomyelitis due to STAT1 LOF variant and primary immunodeficiency disease was significantly improved after anti-tuberculosis treatment: a case report

**DOI:** 10.3389/fped.2025.1626146

**Published:** 2025-09-05

**Authors:** Haiping Ouyang, Di Yang, Zhongliang Wang

**Affiliations:** Department of Orthopedics Children's Hospital of Chongqing Medical University, National Clinical Research Center for Child Health and Disorders, Ministry of Education Key, Laboratory of Child Development and Disorders, Chongqing Municipal Health Commission Key Laboratory of Children's Vital Organ Development and Diseases, Chongqing, China

**Keywords:** case report, BCG vaccine disease, systemic multiple osteomyelitis, anti-tuberculosis therapy, primary immunodeficiency disease

## Abstract

**Background:**

This was a rare case where the diagnosis was not obvious during treatment, but the treatment was effective after diagnosis. An infant with recurrent fever was considered for systemic multiple osteomyelitis after two surgical biopsies. After a third operation to take a lymph node biopsy, the patient was finally diagnosed as having disseminated Bacille Calmette-Guerin(BCG) disease caused by BCG vaccination. After diagnosis, the child was effectively treated with anti-tuberculosis therapy.

**Case description:**

A 2-month-old female patient was hospitalized twice for fever and surface mass. The patient underwent a puncture biopsy of the right tibia and a puncture biopsy of the lesion of the right leg respectively. The patient was diagnosed with systemic multiple osteomyelitis. The patient still had recurrent fever after antibiotic treatment. At outpatient follow-up, the patient was found to have primary immunodeficiency disease with STAT1 LOF mutation. When the child was one year and one month old, she was hospitalized again with a fever. The patient underwent a third operation, a biopsy of the left axillary lymph node. The pathological results suggested granulomatous inflammation, which was considered tuberculosis. The child was diagnosed with disseminated BCG vaccine disease. After 16 months of oral treatment with isoniazid, rifampicin, ethambutol,and levofloxacin, the child's condition was significantly improved.

**Conclusions:**

The performance of multiple surgical biopsies is crucial in cases of infants presenting with recurrent fever and widespread bone destruction, as well as in children diagnosed with primary immunodeficiency disease, particularly when the available etiological tests offer limited diagnostic evidence.

## Introduction

The bacille Calmette-Guerin (BCG) vaccine was first given orally to children in 1921 and remained the only vaccine approved to prevent tuberculosis (1). BCG vaccine is widely used in countries where tuberculosis is highly endemic and all newborns receive a single intradermal injection either immediately after birth or later in infancy (2–4). Reported complications of BCG vaccination include local ulcers, scarring, local injection site abscesses, and lymphadenitis (5). Systemic disseminated BCG disease is a recognized but rare consequence of BCG vaccination and has traditionally been seen in children with severe immune deficiencies. BCG osteomyelitis, which is not common, can sometimes leave patients with long-term sequelae, including limb length differences or kyphosis (6). The occult nature of BCG disease osteomyelitis makes it difficult for clinicians to detect it early. In terms of suspected BCG osteomyelitis findings, the most common symptoms were swelling (77.5%), tenderness (54.9%), palpable mass (51.4%), fever (21.1%), redness (33.8%), and local fever (22.5%) (7). However, in BCG osteomyelitis, acid-fast Bacillus stain is usually negative, while Mycobacterium bovis culture is positive (8). Polymerase chain reaction (PCR) is a useful tool because it enables rapid detection of mycobacterium tuberculosis complexes before tissue culture results are available. So we can do early detection, and early treatment. However, for children with impaired immune function, it is sometimes difficult to make a clear diagnosis, leading to a gradual worsening of the disease. A total of 485 congenital immunodeficiency disorders have been identified. Its clinical manifestations are increased susceptibility to infection, autoimmune, autoinflammatory diseases, allergies, bone marrow failure, or malignancy (9). In the case we reported, the patient was diagnosed with systemic multiple osteomyelitis following two surgical needle biopsies. Subsequently, primary immunodeficiency was identified through genetic testing. The patient then underwent a third operation, and postoperative pathology indicated tuberculosis as the underlying cause. Her primary condition was determined to be disseminated BCG disease, resulting in BCG osteomyelitis. Following anti-tuberculosis treatment, the patient's systemic symptoms improved, and bone regeneration gradually occurred. In this case, only the PPD test was positive for tuberculosis.

Multiple biopsies are necessary in infants with unexplained fever and systemic multiple osteomyelitis when etiological tests are negative. For complex diseases, only when we know the cause of the disease, can we make the right treatment plan, to shorten the damage of the disease to the patient's health. The patient's written informed consent was obtained for publication of this case report.

## Case presentation

**Figure 1:**
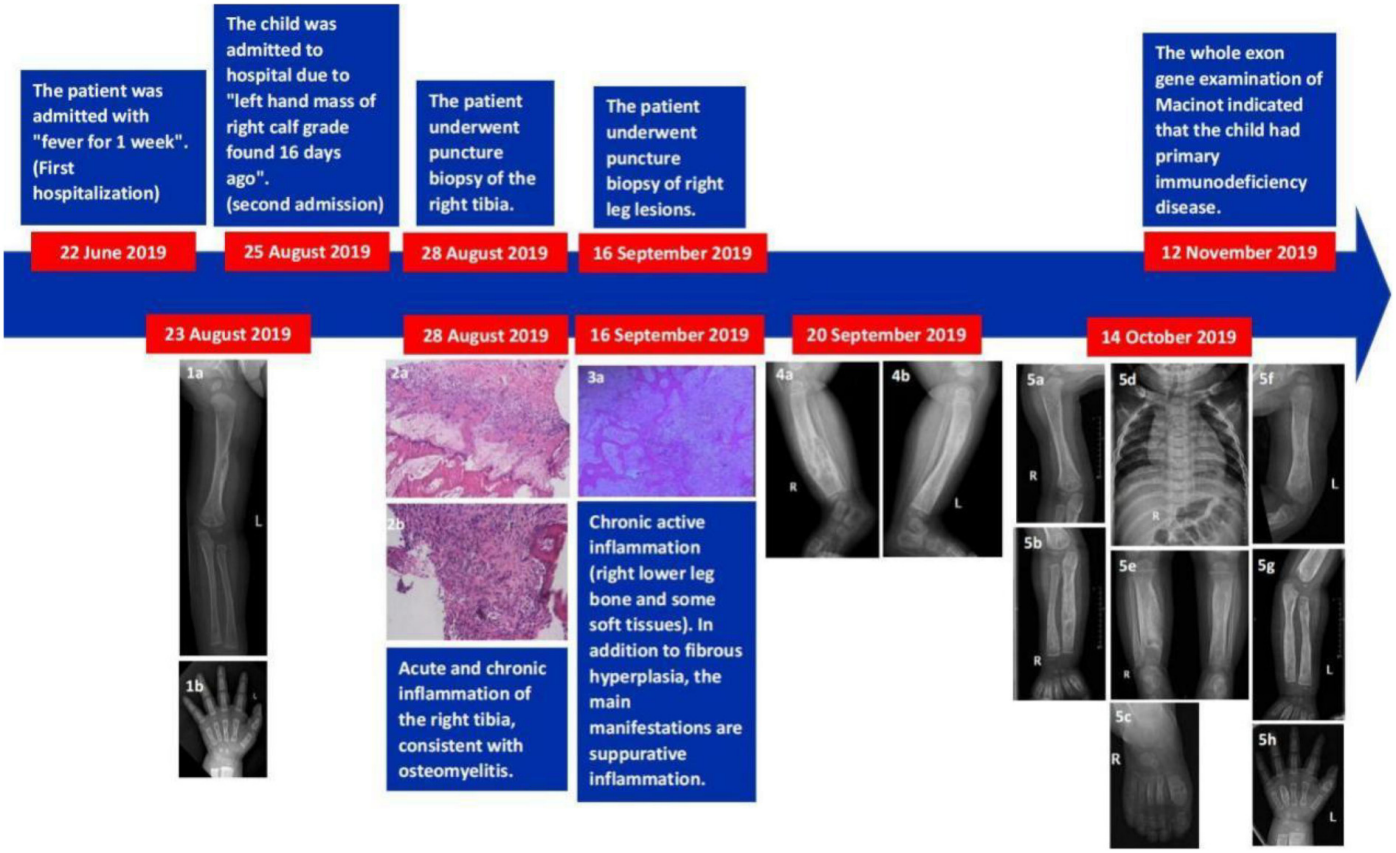
Illustrative summary of the case. On August 23, 2019, outpatient x-rays revealed bone changes in the middle part of the left humerus and the proximal phalanx of the left thumb **(1a,1b)**. On August 28, 2019, pathological results of the first surgical biopsy indicated acute and chronic inflammation, consistent with osteomyelitis **(2a,2b)**. On September 16, 2019, the pathological results of the second surgical biopsy indicated chronic active inflammation, mainly manifested as suppurative inflammation **(3a)**. On September 20, 2019, a reexamination x-ray revealed bone changes in bilateral tibia, with the right tibia being more obvious **(4a,4b)**. On October 14, 2019, the re-examination x-ray showed multiple bone changes **(5a-5h)**.

On June 22, 2019, a 2-month-old girl was hospitalized in the cardiovascular department of our hospital due to “intermittent fever for 1 week”. The fever usually occurred in the afternoon, and a few rashes appeared on the trunk and limbs after the body temperature was normal. The rash then resolved automatically, and her fever returned to normal after 2–3 days. She was her mother's first child and was born naturally with a birth weight of 4 kg. She is breastfed after birth. She was given the BCG vaccine after birth. The child's parents were in good health, but one of the child's uncles had primary immunodeficiency. After admission, the patients were mainly given anti-inflammatory treatment, including cefazoxime and meropenem. The patient was discharged after 4 days without fever. On August 25, 2019, the patient was admitted to the orthopedic department “16 days after the discovery of a mass in his right calf and left thumb.” Before admission, outpatient x-rays revealed bone changes at the distal metaphysis of the right tibia and the middle of the left humerus. When she was admitted to the hospital, the physical examination showed that several lymph nodes of different sizes could be touched in her bilateral neck, armpit, and groin area, which were soft and had no obvious tenderness. The left thumb and the metacarpophalangeal joints of the second finger of the left hand were swollen significantly, and the skin temperature was normal without obvious tenderness. The skin of the distal tibia of both lower limbs was raised, and the skin temperature was normal without obvious tenderness. After admission, the blood test results of the child showed that the white blood cell count was 20.17*10^9/L, CRP was 30 mg/L, erythrocyte sedimentation rate was 84 mm/1 h, and PCT was 0.107 ng/ml. The blood culture results were negative. On August 28, 2019, the patient underwent a puncture biopsy of the right tibia. The pathological report of the child suggested acute and chronic inflammation, consistent with osteomyelitis. Then she was given ceftiamidine to treat the infection. After surgery, the swelling of the patient's right lower limb worsened, and a biopsy of the right calf lesion was performed on September 16, 2019. The postoperative pathological report indicated chronic active inflammation, except fibroplasia, which was mainly characterized by suppurative inflammation. The child was then given ceftiamidine to fight the infection. The swelling of her right lower limb was significantly better than before, and she was discharged after a week without a fever. On October 14, 2019, the patient was followed up in the outpatient department of our hospital. x-rays showed more bone changes consistent with multiple osteomyelitis in the middle of the left humerus, the proximal phalanx of the left thumb, the distal end of the left radius, the fourth metacarpal bone of the left hand, the bilateral tibia, the right fibula, some ribs, the first and second metatarsal bones of both feet, and the third metatarsal bone of the left foot. On November 12, 2019, the patient underwent a complete Maikino exon examination during outpatient follow-up in our hospital, and the examination results indicated that the patient had primary immunodeficiency disease accompanied by STAT1 LOF gene mutation (Figure 1). It was highly correlated with the clinical phenotype of the children, and the variant was *de novo* by family analysis. Follow-up visits were conducted in the outpatient department of our hospital on January 2, 2020, and April 2, 2020. x-rays showed that bone lesions of both hands, feet, scapula, clavicle, and pelvis increased. Multiple bone destruction of skull and limbs.

On May 13, 2020, the patient was hospitalized in the Rheumatology and Immunology Department of our hospital due to “intermittent fever for 11 months”. Upon admission, the patient had enlarged lymph nodes of varying sizes in the neck, armpit, and groin area, and multiple joints of the hands and feet were swollen. After admission, the possibility of tuberculosis was considered, and relevant examinations were carried out, such as blood culture, bacterial/fungal smear, acid-fast bacillus smear, sputum smear, mycobacterium tuberculosis, and drug resistance gene determination, tuberculosis interferon assay, PPD test. Only the PPD test results were positive, and the specific results were moderate positive at 24 h (local hard mass diameter 10–15 mm), strong positive at 48 h (local hard mass diameter ≥15 mm), and strong positive at 72 h (local hard mass diameter ≥15 mm). The results of other items were negative. During admission, the patient underwent magnetic resonance imaging (MRI) of both legs and feet. The results showed abnormal signals of both tibia-fibula and foot bones, mainly granuloma formation, accompanied by edema of surrounding soft tissues. The possibility of chronic low-virulence infection was considered comprehensively. On May 20, 2020, the patient underwent a third operation, a biopsy of the left axillary lymph node. The postoperative pathological results suggested granulomatous inflammation, and the possibility of tuberculosis was considered, and the DNA-PCR results of tuberculosis bacilli were negative. At the same time, immunohistochemistry was added to the first and second surgical biopsy specimens, and the results showed Langerin (-), CD1a (-), CD68 (+), acid-fast staining (-), PSA staining (-), no fungal spores and mycelia, and tuberculosis DNA-PCR (-). After multidisciplinary consultation and discussion in the whole hospital, combined with the results of the type of primary immunodeficiency disease of the patient's susceptibility to mycobacterium tuberculosis and the involvement of multiple organs in the whole body, the child was finally diagnosed as disseminated BCG vaccine disease. May 27, 2020 Start of anti-tuberculosis treatment. Isoniazid 100 mg, rifampicin 100 mg, ethambutol 0.1875g and levofloxacin 100 mg were given orally once a day. The patient was discharged after the swelling of the hands and feet decreased and the temperature returned to normal.

**Figure 2:**
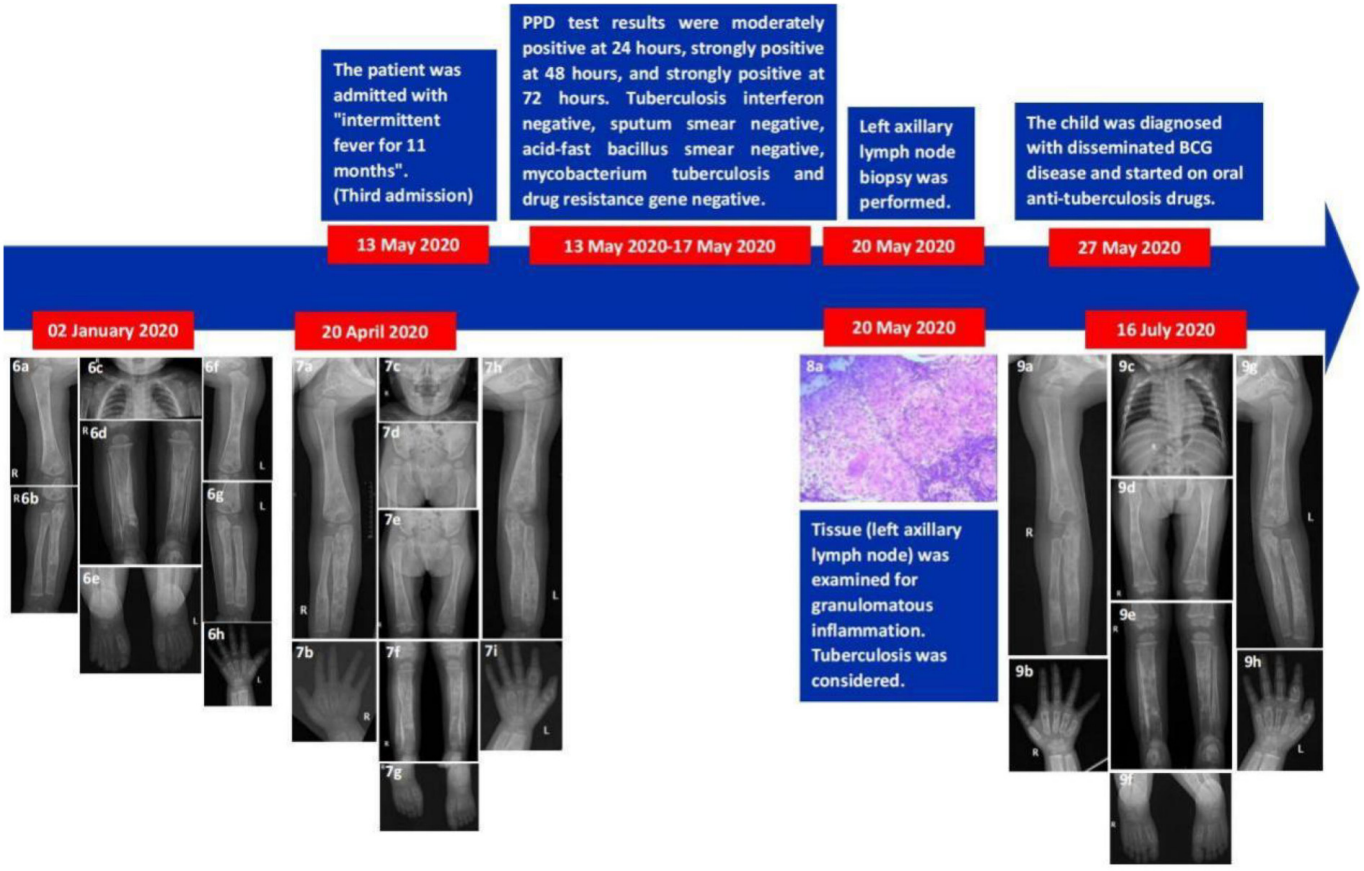
Illustrative summary of the case. On January 2, 2020, the re-examination x-ray indicated multiple bone destruction **(6a–6h)**. On April 20, 2020, the re-examination x-ray still indicated multiple bone destruction **(7a–7i)**. The pathology of the biopsy taken from the third operation on May 20, 2020, indicated granulomatous inflammation, considering tuberculosis **(8a)**. On July 16, 2020, an x-ray reexamination in the clinic indicated that the extent of bone destruction in multiple parts of the body was reduced **(9a–9h)**.

After discharge, the patient was given oral antituberculosis drugs for 16 months and was followed up regularly in the outpatient department of our hospital. On August 21, 2020, the outpatient doctor adjusted the dosage of isoniazid 0.125 g, rifampicin 0.15 g, ethambutol 0.2 g, levofloxacin 0.15 g. On February 17, 2021, the outpatient doctor again adjusted the dosage of isoniazid 0.15 g, rifampicin 0.15 g, ethambutol 0.2 g, and levofloxacin was discontinued. On September 27, 2021, the patient began to stop taking anti-tuberculosis drugs (Figure 2). On July 16, 2020, October 15, 2020, March 4, 2021, and September 9, 2021, the patient was re-examined in the outpatient department of our hospital. x-ray results showed that multiple bone destruction of the long bones of the right hand, feet, and limbs was significantly repaired. The patient's last outpatient review was on September 9, 2022. x-ray images showed that multiple bone lesions in the long bones of the left hand, feet and limbs were repaired compared with the previous images (September 9, 2021). The maxillofacial bones showed no obvious abnormality.

**Figure 3:**
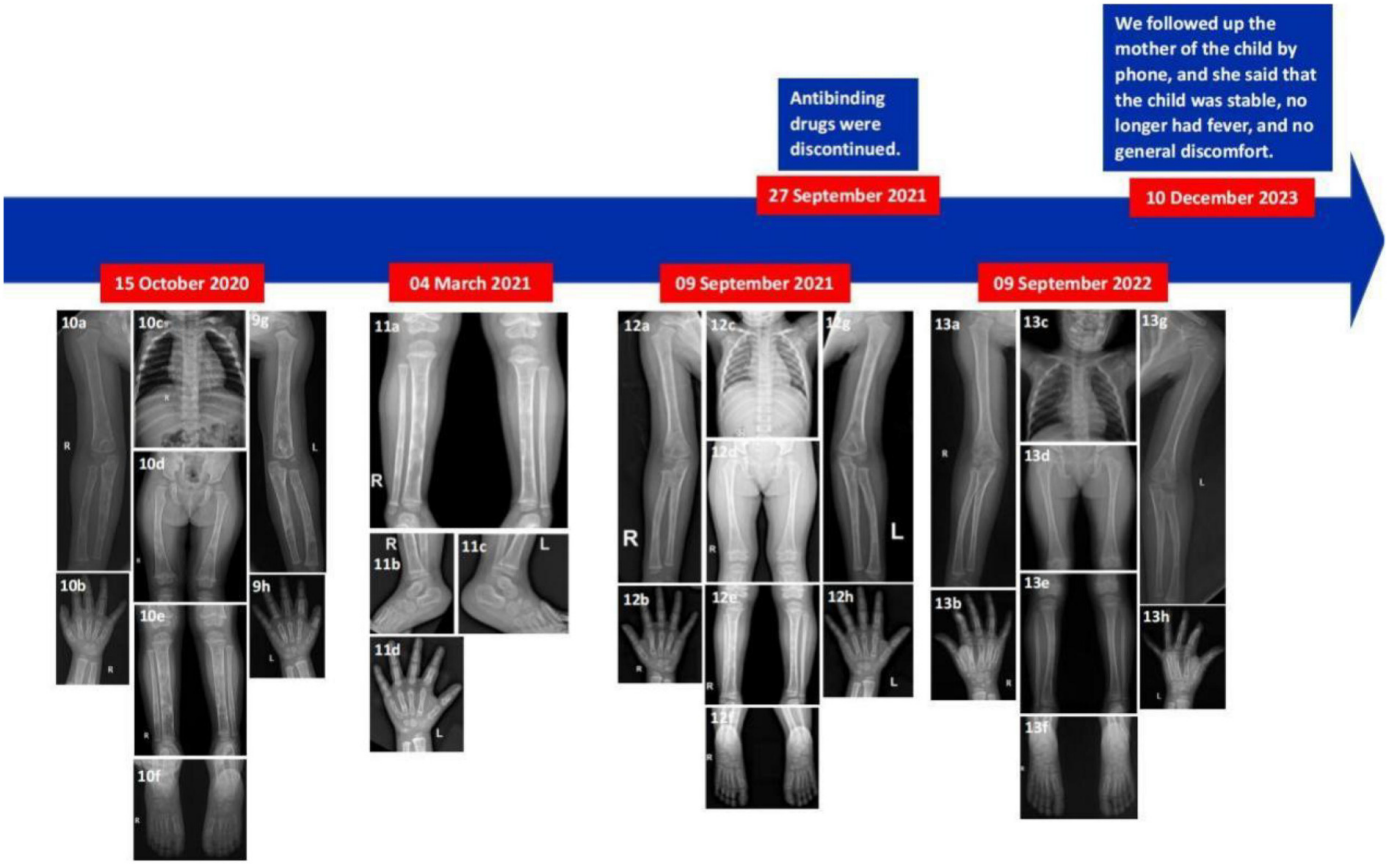
Illustrative summary of the case. On October 15, 2020, an x-ray reexamination in the clinic indicated that multiple bone destruction in the whole body was further repaired than before **(10a–10h)**. On March 4, 2021, reexamination x-rays indicated improved bone destruction of the left hand and bilateral tibia and fibula **(11a–11d)**. On September 9, 2021, re-examination x-rays indicated that multiple bone damage in the whole body was significantly repaired **(12a–12h)**. On September 9, 2022, a re-examination x-ray showed that multiple bone lesions in the whole body were repaired **(13a–13h)**.

After anti-tuberculosis treatment, the body temperature of the child was normal and there was no recurrence of fever. The swelling of the left hand was significantly improved, and x-rays of the clinic showed significant improvement in the bones. During the period of medication, the blood regularity of the child has been checked, and the liver and kidney function and blood routine are followed up, and the results are not obviously abnormal. On December 10, 2023, we called the family of the child and learned that the child was in a stable condition without fever, obvious mass, and pain in the bones of the whole body (Figure 3). There were no obvious abnormalities in her growth and intelligence.

## Discussion

Exclusively in people with congenital or acquired immunodeficiency disease who are unintentionally immune (10). In one study, 9 out of 15 children with disseminated BCG had primary immunodeficiency disease including severe combined immunodeficiency, chronic granulomatous disease and cell-mediated immune defect, and HIV infection (11). One previous case, similar to our study, described a 9-month-old girl who was given the BCG vaccine after birth and was found to have severe combined immunodeficiency. She ended up with widespread osteomyelitis. The disease deteriorated rapidly, with diffuse intravascular coagulation (DIC), total organ failure, and death (12). The child in our case is the lucky one. Because the child was given anti-tuberculosis treatment after the diagnosis of disseminated BCG vaccine disease, the condition was significantly improved. There are currently no randomized controlled trials for BCG osteitis that serve as treatment guidelines for anti-TB drugs (7). The data in the systematic review concluded that a combination of isoniazid and rifampicin could be used as an initial treatment option (13). In this previous study, patients with disseminated BCG disease were treated with isoniazid, rifampicin, ethambutol, and streptomycin (11). In our case, the anti-tuberculosis drugs used were isoniazid, rifampicin, and ethambutol, with the addition of levofloxacin. According to current WHO guidelines (updated in October 2016), fluoroquinolones, namely moxifloxacin, Gatifloxacin, and levofloxacin, are the most valuable second-line anti-tuberculosis drugs (14). The fluoroquinolone target in Mycobacterium tuberculosis is DNA gyrase (topoisomerase II) (15). Inhibition of DNA gyrase disrupts bacterial DNA synthesis causing rapid cell death (16). The use of fluoroquinolones in children is limited because of their potential to cause joint disease in young animals (17–19). However, most reported adverse reactions to fluoroquinolone use in children have been mild, require little or no intervention, and are reversible with drug discontinuation (20).

There are some diseases with clinical manifestations similar to BCG osteomyelitis. One of them is Infantile cortical hyperostosis. It is characterized by unusual irritability, soft tissue swelling, and cortical osteosis of multiple bones (21). Some patients develop fever and anemia (22). Affected bones include the mandible, tibia, ulna, clavicle, scapula, ribs, humerus, femur, fibula, skull, ilium, and metatarsals (21). Because the symptoms are very similar to this case, the etiology of Infantile cortical hyperostosis is negative, and their radiological characteristics are different. Infantile cortical hyperostosis is the formation of new bone in the periosteum and the thickening of the cortex, which will eventually reshape and return to normal appearance (21). Another similarity to BCG disease is chronic recurrent multifocal osteomyelitis. Chronic recurrent multifocal osteomyelitis (CRMO) is a rare autoinflammatory disease characterized by recurrent and remitting episodes of pain associated with the presence of sterile bone inflammatory lesions (23). The disease can affect all bones, but the lesions usually occur in the metaphysis and epiphysis of the long bones and are more common in the lower extremities (24–27). CRMO is primarily a diagnosis of exclusion (28). Malignant tumors need to be ruled out first, followed by infections and other inflammatory diseases (29). The pathophysiological feature of tuberculosis is caseous necrosis, which is thought to be caused by macrophage death mediated by mycobacterium tuberculosis (30–32). In our case, the pathology report of the third surgical biopsy showed granulomatous inflammation, but no definite caseous necrosis. The inaccuracy of tissue localization during the three operations may be one possible reason for not obtaining the lesion area containing caseous necrosis. PID is a group of genetic disorders that result in an impaired cellular and/or humoral immune response, with phagocyte defects leading to a high susceptibility to infection, particularly to mycobacteria (33). This may be another reason why no caseous necrotic tissue was found in the three surgical biopsies.

In the course of treatment, the child was found to have primary immunodeficiency disease through genetic testing, accompanied by STAT1 LOF gene mutation. As described in a systematic review, the most common manifestation in patients with STAT1 LOF mutations is Mendelian susceptibility to mycobacteriosis (MSMD), and BCG strains are the most common pathogenic agent of MSMD in patients with STAT1 LOF. Osteomyelitis is relatively common in patients with STAT LOF mutations and most cases are caused by mycobacteria (34). Therefore, we need to be alert to BCG osteomyelitis when we encounter unexplained fever and bone destruction in infants with primary immunodeficiency disease associated with STAT1 LOF mutation. A systematic review of BCG osteomyelitis described that surgical intervention was mentioned in less than 25% of patients, and the authors concluded that surgical intervention for diagnostic purposes was helpful (13). The patient's tuberculosis interferon test results were negative due to the fact that our hospital's tuberculosis interferon test specifically detects Mycobacterium tuberculosis in humans, while children are vaccinated with BCG vaccine containing Mycobacterium bovis. This idea of surgical intervention for definitive diagnosis is consistent with us. When the cause of the disease is unclear, specimens can be obtained with minimal surgical trauma to assist in diagnosis and guide treatment. In our case, there was an unknown cause of fever and multiple bone destruction throughout the body. However, there is no evidence for a definitive diagnosis of BCG disease, so surgical biopsy is a crucial option. The examination of immune function should be enhanced postnatally for newborns with a family history of primary immunodeficiency disease to prevent the occurrence of disseminated BCG disease following vaccination.

Similar to most case reports, our study was a retrospective study and the number of case reports was very limited, which was sometimes difficult to popularize. Another shortcoming of this case report is that it took a long time to identify the primary disease of the child, so that the disease of the child continued to worsen before a definitive diagnosis was made. This is also closely related to children with primary immunodeficiency disease. The clinical symptoms of children with immune deficiency associated with mycobacterium tuberculosis infection are not typical, the positive rate of PPD and *γ*-interferon release assay detection is low, and it is easy to misdiagnose and miss diagnosis (35). One of the advantages of this study is that the patient's history is clear, and the follow-up time is long, and the patient's condition is significantly improved after a clear diagnosis. Although a STAT1 loss-of-function (LOF) variant highly correlated with the patient's clinical phenotype was identified in this case through whole-exome sequencing, the inability to access the initial testing report precluded direct comparison with previously reported variants. This limitation hinders confirmation of whether the variant is novel or a known variant associated with Mendelian susceptibility to mycobacterial diseases (MSMD). Furthermore, the absence of specific nomenclature may compromise its comparability with other studies and integration into variant databases. Future studies should prioritize determining the precise nomenclature of this variant through additional genetic analyses to facilitate its classification within the MSMD literature and validate its pathogenicity.

The performance of multiple surgical biopsies is crucial in cases of infants presenting with recurrent fever and widespread bone destruction, as well as in children diagnosed with primary immunodeficiency disease, particularly when the available etiological tests offer limited diagnostic evidence. Biopsies are conducted to accurately identify the underlying cause of the disease for precise treatment interventions.

## Data Availability

The original contributions presented in the study are included in the article/Supplementary Material, further inquiries can be directed to the corresponding author.
